# Advanced Research in Glaucoma: Treatment and Postoperative Approaches

**DOI:** 10.3390/jpm13060895

**Published:** 2023-05-25

**Authors:** Kazuyuki Hirooka

**Affiliations:** Department of Ophthalmology and Visual Science, Hiroshima University Graduate School of Biomedical Sciences, 1-2-3 Kasumi, Minami-ku, Hiroshima 734-8551, Japan; kazuyk@hiroshima-u.ac.jp; Tel.: +81-82-257-5247; Fax: +81-82-257-5249

The principal proven treatment methods for glaucoma management almost exclusively focus on lowering the intraocular pressure (IOP). These methods use drugs, laser therapy, or surgery. When the IOP is not reduced to target levels after initial medical or laser treatments, surgery should be considered. Moreover, despite maximum medical therapy in some patients, there is a continued deterioration of the optic nerve observed. Investigations of IOP-lowering medication, laser therapies, or incisional surgery are needed to ensure both the efficacy and safety of these procedures. Furthermore, not only glaucoma patients but also clinicians benefit from the results of these types of studies, with the accumulation of these results helping to improve the quality of life of glaucoma patients. In this Special Issue, the latest visual field tests, IOP-lowering medications, and glaucoma surgery results have been provided by many researchers and research groups.

An invaluable tool that has been utilized to detect and monitor glaucoma associated visual function loss is visual field testing using semiautomated perimeters. However, it is crucial to determine whether the test was reliably performed prior to interpreting the visual field test results. When monitoring glaucoma progression, evaluation of the reliability indices is critical. A higher variability in visual field testing is associated with severe stages of glaucoma. Another factor—and perhaps the most important factor—that can affect the results of visual field tests is aging. Although higher false negatives were significantly associated with older age, older age was not found to be related to fixation losses or false positives [1]. In our current study, when examining severe visual field defects, especially in older patients, similar to other studies, we found that choosing the 10-2 program versus the 30-2 program was effective in reducing the fixation losses.

When treating these patients with pharmacological agents, prostaglandin analogues, β-blocker, carbonic anhydrase inhibitors (CAI), α_2_-adrenergic agonists, and Rho-kinase inhibitors are commonly administered. However, when insufficient outcomes are found after monotherapy, the use of a combination therapy with multiple IOP-lowering medications is required in order to achieve successful management of glaucoma. At the present time, instead of using a β-blocker, brinzolamide/brimonidine and brimonidine/ripasudil have been utilized for fixed-combination glaucoma therapy. As compared to using a concomitant dosing of two separate medications, there are some advantages associated with the utilization of a fixed-combination medication. Onoe et al. examined the switching of patients from brinzolamide 1% or brimonidine 0.1% to brinzolamide/brimonidine fixed-combination ophthalmic suspension (BBFC) and then investigated the efficacy, safety, and satisfaction in these subjects [2]. Changes in treatment satisfaction were assessed by the Treatment Satisfaction Questionnaire for Medication-9 (TSQM-9). After switching to BBFC, results indicated that there was a significant decrease in the mean IOP [2]. After switching to BBFC from brinzolamide 1% or brimonidine 0.1%, results showed that the patients were indeed aware of the effectiveness of this change in their therapy [2]. Thus, adherence could potentially be improved by using fixed-combination medication. Various factors, such as the appearance of a new medication class, have led to changes in the trends in IOP-lowering medication prescriptions. An examination of prescription trends in 2019 showed that the number of doses of prostaglandin FP2α agonist, followed by the fixed-combination of β-blocker/CAI and α_2_-adrenergic agonist, were the largest according to the National Database of Health Insurance Claims and Specific Health Checkups of Japan Open Data [3]. Moreover, age, gender, and the use of generic medications were also found to be associated with these IOP-lowering medication prescriptions. The findings from these previous studies provide up-to-date, real-world information on IOP-lowering medication prescriptions.

There is a high risk of the development of glaucoma in uveitis patients. Furthermore, as the control of the IOP by IOP-lowering medications is hard to achieve in patients with uveitic glaucoma, these patients will sometimes require glaucoma surgery. It has been reported that there was a lowering of the IOP in post-uveitic patients with moderate glaucoma after undergoing removal of the internal wall of the Schlemm’s canal with the TrabEx+ device, or a minimally invasive surgical procedure [4]. For the phacoemulsification procedure, TrabEx+ utilizes a handpiece with an irrigation and aspiration system that can be adapted for each machine. When using this procedure, there was a 20% or more reduction of the IOP from baseline in all of the patients observed during the follow-up period [4]. After glaucoma surgery, however, there is a great deal of concern with regard to corneal endothelial cell density (CECD) loss, especially when there is implantation of a glaucoma drainage device. Moreover, as CECD loss can be affected by ocular inflammation, in patients with uveitic glaucoma, it is important that CECD changes be investigated after implantation of a glaucoma drainage device. In patients with uveitic glaucoma treated by the ciliary sulcus placement of the Ahmed glaucoma valve (AGV), a previous study found that the mean CECD was 2431 ± 368 cells/mm^2^ at the preoperative baseline and 2361 ± 391 cells/mm^2^ at 12 months, with a deduction rate of 2.73 ± 9.29% [5]. The mean preoperative IOP was 28.7 ± 9.6 mmHg, and at 12 months postoperatively, the IOP was significantly decreased to 13.3 ± 4.5 mmHg [5]. Based on the results of this study, it was concluded that the implantation of the AGV tube into the ciliary sulcus was able to provide stable IOP control, and that in eyes with uveitic glaucoma, the CECD loss was moderate.

As Guest Editor, I would like to thank all the authors and reviewers for their contributions to this Special Issue and for the high quality of the papers that were submitted. I hope the novel findings presented here will help facilitate further developments and lead to a refinement of personalized medicine in the treatment of glaucoma.
